# Differences of Psychopathology of Psychiatric Disorders in Adult Patients with Intellectual Disability

**DOI:** 10.1192/j.eurpsy.2022.176

**Published:** 2022-09-01

**Authors:** M. Musalek

**Affiliations:** Sigmund Freud University, Faculty of Medicine, General Psychiatry, Vienna, Austria

**Keywords:** phenomenology, Psychopathology, daily life overload syndrome

## Abstract

Intellectual disability is a mental disorder which appears during the developmental period in childhood and continues life-long. It is characterized by intellectual deficits on the one side and maladaptive social functioning on the other. The intellectual deficits include problems in abstract and logic thinking, problem solving, reasoning, learning from experience and judging. The deficits in adaptive functioning appear in problems in social communication and participation as well as in mental overload phenomena caused by daily life activities. This overload often results in psychopathological features and psychosocial reactions which are usually observed in Burn-out-Syndromes as e.g. general exhaustion (depletion of primarily reduced mental resources), additional reduced efficacy (accompanied by feelings of insufficiency and poor self-esteem), and most typically feelings alienation (towards intrinsically familiar situations, towards relatives and friends and at least also against oneself ) typically accompanied by irritability and dysphoria. Treatment programs therefore should not only focus on intellectual deficits but should include also the multiple sufferings from psychopathological phenomena and psychosocial effects of such daily life overload syndromes.

**Disclosure:**

No significant relationships.

